# Isolated free intraperitoneal fluid in young male after blunt abdominal sport trauma: two case reports from the World Cup 2022

**DOI:** 10.1093/jscr/rjad561

**Published:** 2023-10-14

**Authors:** Abubaker Al-Aieb, Husham Abdelrahman, Sandro Rizoli, Ayman El-Menyar, Syed Nabir, Ahmad Kloub, Hassan Al-Thani

**Affiliations:** Department of Surgery, Trauma Surgery, Hamad Medical Corporation, P.O. Box 3050, Doha 24144, Qatar; Department of Surgery, Trauma Surgery, Hamad Medical Corporation, P.O. Box 3050, Doha 24144, Qatar; Department of Surgery, Trauma Surgery, Hamad Medical Corporation, P.O. Box 3050, Doha 24144, Qatar; Department of Surgery, Trauma Surgery, Clinical Research, Hamad Medical Corporation, P.O. Box 3050, Doha, Qatar; Department of Clinical Medicine, Weill Cornell Medical College, P.O. Box 24144. Doha, Qatar; Department of Radiology, Hamad Medical Corporation, P.O. Box 3050, Doha, Qatar; Department of Surgery, Trauma Surgery, Hamad Medical Corporation, P.O. Box 3050, Doha 24144, Qatar; Department of Surgery, Trauma Surgery, Hamad Medical Corporation, P.O. Box 3050, Doha 24144, Qatar

**Keywords:** intraperitoneal, free fluid, healthy male, blunt trauma, abdomen, sport injury

## Abstract

The presence of isolated intraperitoneal free fluid (IFIPF) indicates the presence of mesenteric, organ, or bowel injury, which necessitates surgical exploration. The advances in computerized tomographic scanning (CT scan) allow even smaller amounts of IFIPF being detected. However, the clinical significance of IFIPF following blunt abdominal trauma remains not well-studied. Moreover, IFIPF is an unexpected condition in healthy male in the absence of mesenteric or organ injury on abdominal imaging. Herein, we presented two cases with IFIPF detected by CT scan in two healthy football male players during the World Cup 2022. The two players were managed conservatively and rejoined safely their football team during the same competition.

## Introduction

The World Cup 2022 was one of the most impressive tournaments. One of Qatar’s expanded healthcare investments in the emergency services, such as a unique Trauma & Emergency specialized center, a dedicated trauma surgery team, emergency medical and paramedical teams, as well as prehospital medical services. These provisions aimed to ensure optimal healthcare the visitors of Qatar, athletes, as well as the citizens and country residents.

Trauma surgery at Hamad Medical Corporation is a specialized field of medicine that focuses on treating all trauma patients through a team of highly qualified professionals, including surgeons, nurses, and other medical staff. Trauma Surgery, under normal circumstances, receives 250–300 patients per month in the Trauma Resuscitation Unit (TRU) at Hamad General Hospital.

The presence of free intraperitoneal fluid in trauma patients, especially males, without detectable solid organ/mesenteric injury poses challenges for radiologists and trauma surgeons regarding interpretation and management [1]. Enhanced computerized tomography (CT) is the preferred imaging technique for assessing the abdomen and pelvis in individuals with blunt trauma. CT provides valuable information about the extent of injuries to solid visceral organs [2]. CT scan detects both direct and indirect signs of bowel injury, particularly with the advancements in multi-detector CT (MDCT) technology [3, 4]. Accurate identification and characterization of these injuries assist surgeons in decision-making, including surgical exploration.

During the tournament period (1 month), 434 patients were managed in the TRU; 71 of them were visitors and players. Of the 71 patients, 38 were trauma cases. Ten of the trauma cases had traumatic brain injuries, 21 had skeletal and extremity injuries, and seven had torso trauma. Football players with isolated extremity injuries were treated outside the trauma center, in a specialized sports hospital (Aspetar).

Regarding these seven torso trauma cases, two cases were chest trauma. The first one was a football player treated conservatively and discharged on the next day. The second one was a visitor who experienced multiple rib fractures accompanied by pneumothorax and hemothorax, and treated with tube thoracostomy, pain control, and pulmonary physiotherapy, and was discharged after 1 week. Both patients were discharged in good condition with no reported complications.

Five patients suffered blunt abdominal trauma. Among the football players with suspected abdominal trauma, we had three admissions. One of them had a pancreatic trans-section with free fluid based on imaging studies. The other two cases exhibited interesting finding of isolated free intraperitoneal fluid without any injuries of solid organs, bowel, or mesentery.

## Case report 1

**Figure 1:**
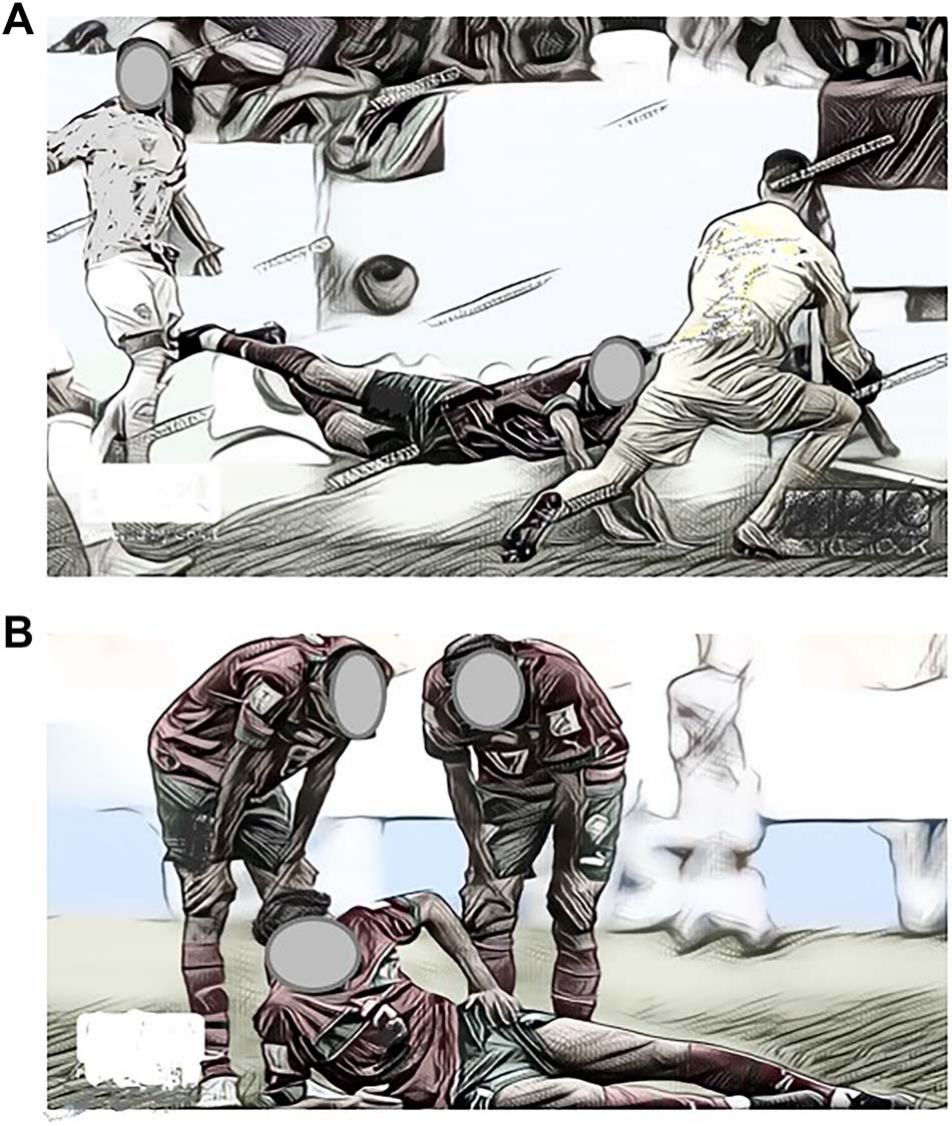
(A, B) A real event photo was captured; however, the color of the uniforms and players faces were modified to mask teams identity.

A 25-year-old football player who fell and landed on his abdomen and left hip while playing, and claimed to have heard a crackling sound above his left hip as depicted in Fig. 1A and B.

He presented to the TRU complaining of abdominal pain. He had mild tenderness in the left iliac fossa and left flank on assessment and a stable pelvis with full-range movement of the left hip.

**Figure 2:**
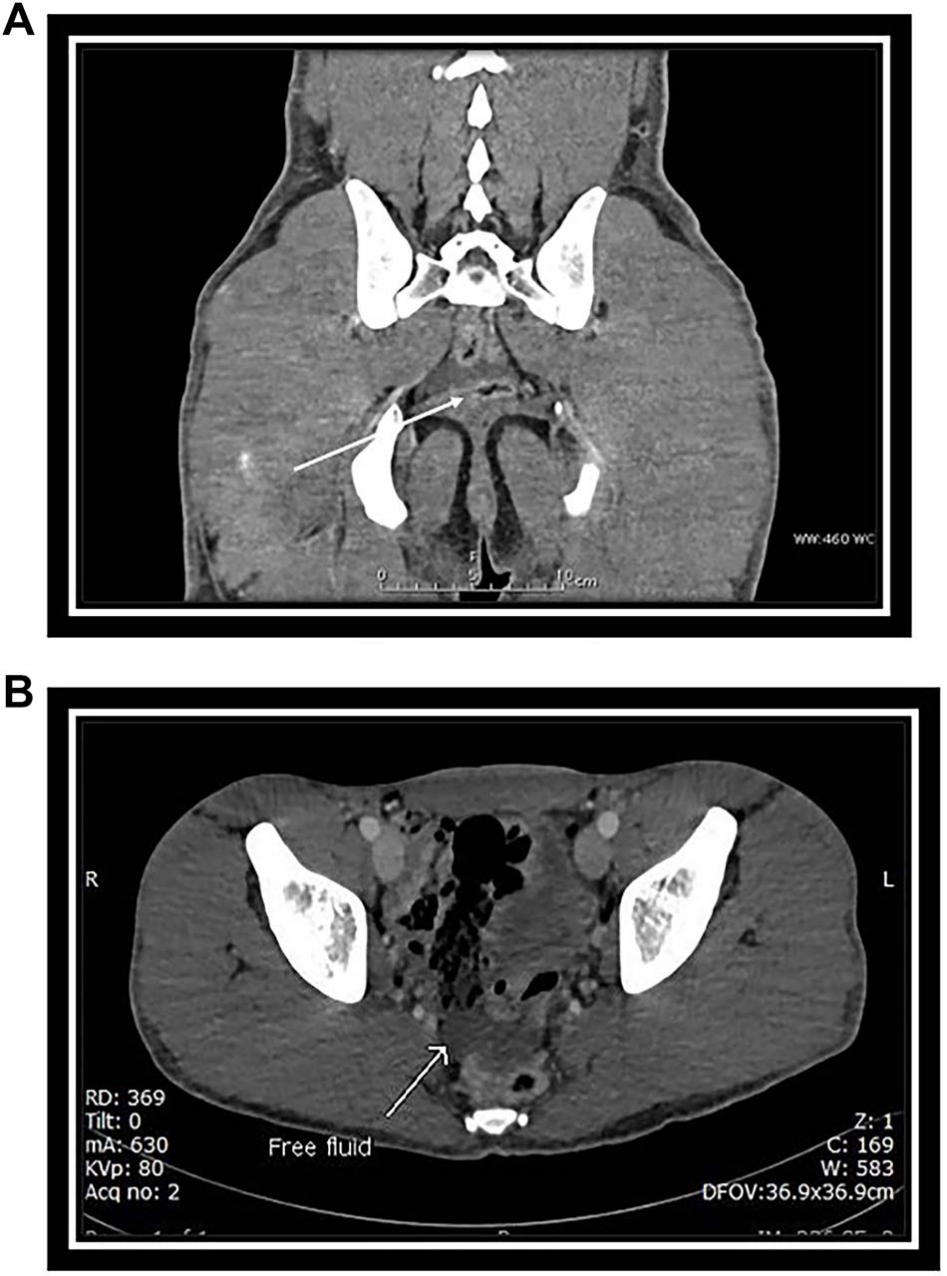
(A) Coronal CT scan section: arrow indicates free fluid in the Douglas pouch. (B) Axial CT scan section: arrow indicates free fluid in the Douglas pouch.

Abdominal CT with intravenous (IV) contrast (Fig. 2A and B) revealed:

1. No solid abdominal organ injuries.
2. Mild free fluid was noted in the pelvis.
3. No abnormal bowel enhancement or bowel dilatation.
4. No free air in the abdomen.
5. The bones were intact.

The player was admitted for one day for monitoring and pain control. He did not develop any complication and was discharged to his camp after 24 h. He was back on the field in his team on the following match.

## Case report 2

**Figure 3:**
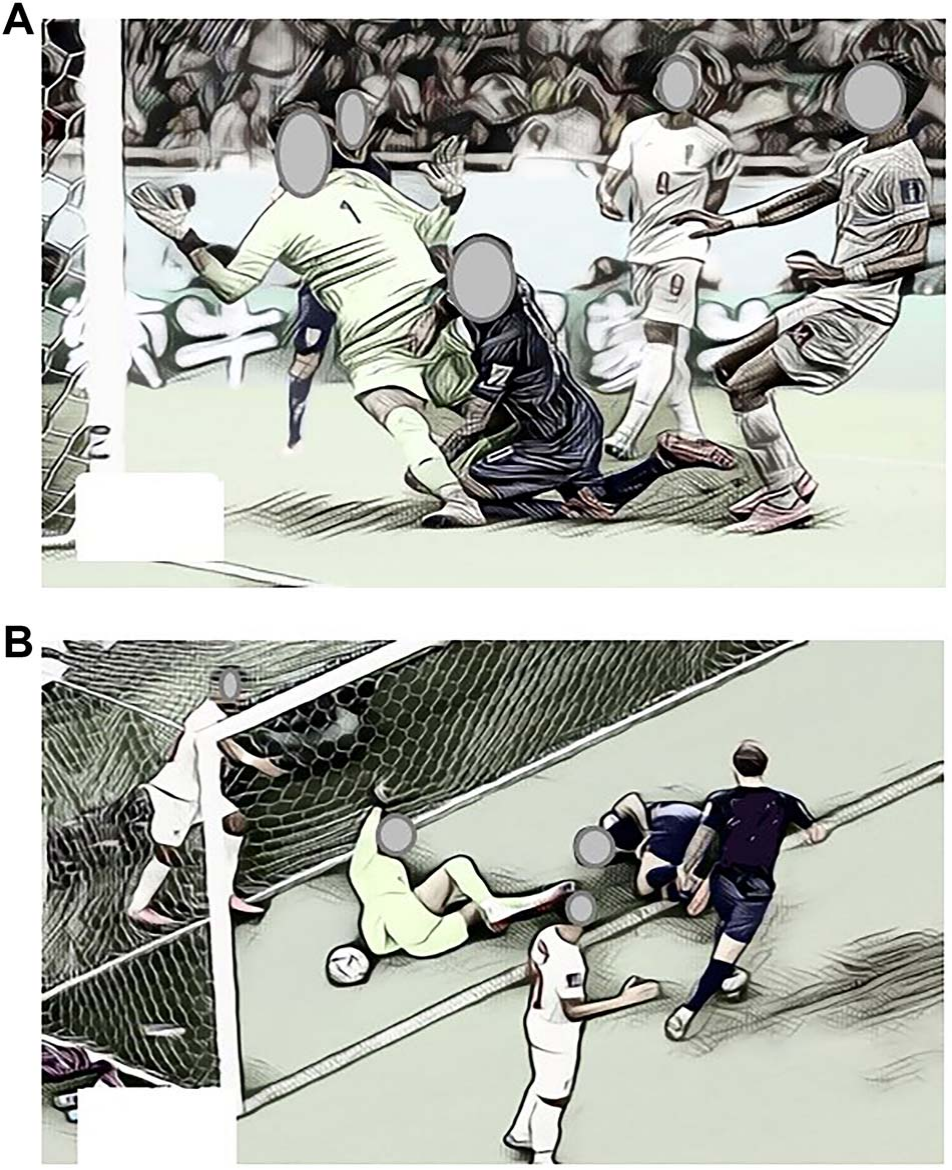
(A, B) A real event was captured; however, the uniform and faces of the players were modified to mask team identity.

A 24-year-old soccer player was kicked by another player’s knee in his abdomen during a match (Fig. 3A and B). He was rescued by the field medical team who decided to take him to our hospital for further evaluation and treatment.

He presented to us with lower abdominal pain. On examination, there was mild tenderness in the suprapubic area. He was voiding freely normal color urine.

**Figure 4:**
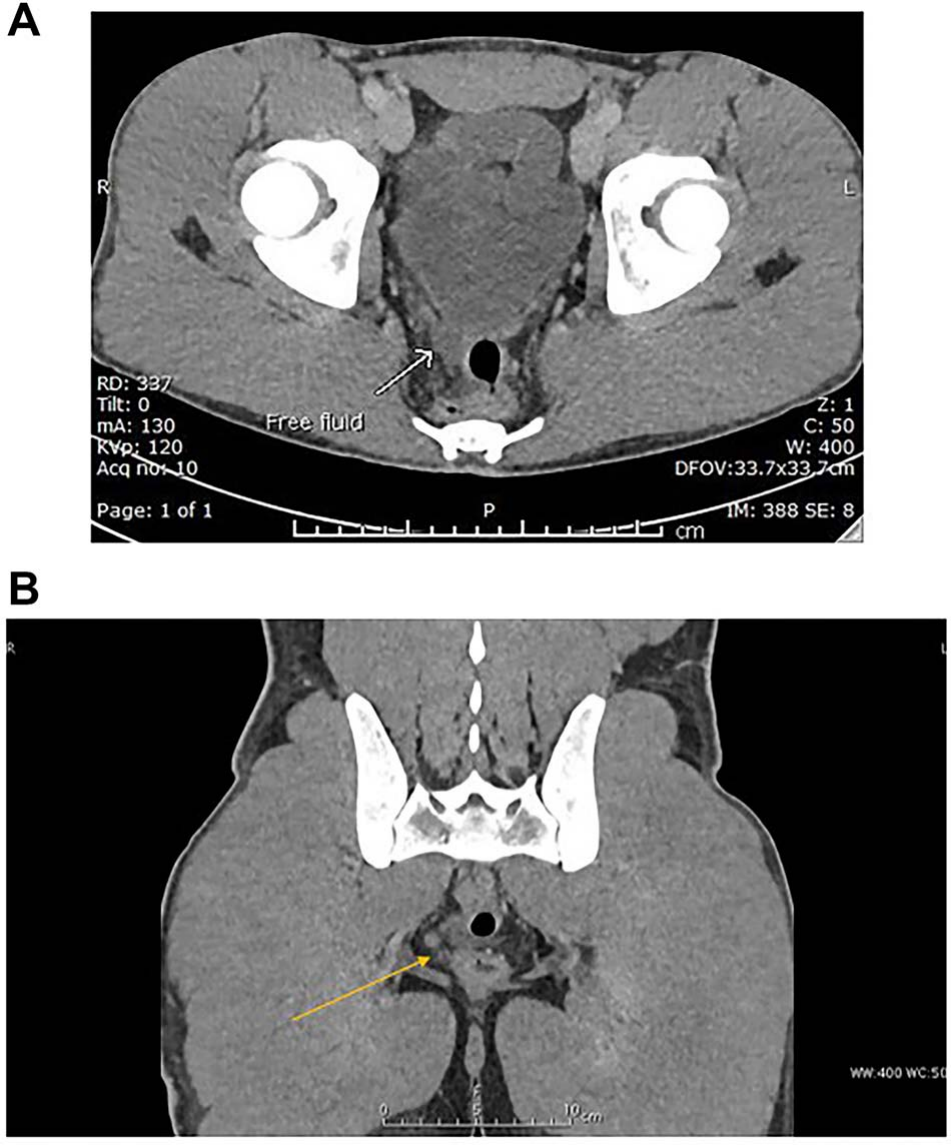
(A) Venous phase axial CT scan showing minimal amount of free fluid in the Douglas pouch (retro-vesical). (B) Venous phase coronal CT scan showing minimal amount of free fluid in the Douglas pouch (retro-vesical).

CT scan of abdomen and pelvis with IV contrast was performed (Fig. 4A and B).

1. Mild free fluid in the pelvis.
2. No intra-abdominal solid organ injury.
3. No free air.
4. No bony injuries.

The patient was admitted for monitoring, but the relevant medical team decided to take over the patient’s care and monitor him at the camp’s medical unit/clinic.

The patient did well. Four days later, he participated in his team’s subsequent match in good physical condition with no reported surgical intervention in the camp medical facility.

## Discussion

Football is one of the common sports with high injury rate [5]. The abdomen is an area of the body that is not well protected, especially during competitive sports like football. In addition to lacking a protective structure, its sensitivity to injury is heightened by its size, intermediate location on the body, and proportion to the body’s surface area.

In nearly all athletic events, injury to the abdomen is a potential hazard, and recognizing the signs of serious damage is imperative. However, with the proper education and training, these injuries can be efficiently treated both on and off the field [6].

The presence of free intraperitoneal fluid in blunt trauma raises concerns of abdominal organ or mesentery injury. However, the clinical significance of isolated free fluid following blunt abdominal trauma remains unclear [7]. Isolated free intraperitoneal fluid is unexpected condition in healthy male.

Advances in imaging technology have made MDCT scan the modality of choice for evaluating blunt abdominal trauma, and its sensitivity to detect small amounts of free intraperitoneal fluid is progressively increasing [4]. Isolated intraperitoneal free fluid (IFIPF) in blunt trauma may or may not represent a significant injury, and this creates a diagnostic dilemma in determining appropriate treatment for these patients [1]. Three to five percent of blunt trauma patients have IFIPF on MDCT [1, 2, 4, 8]. A study on blunt abdominal trauma found that isolated free fluid on CT is 98% sensitive and 96% specific for true isolated free fluid [8]. Moreover, they concluded that the finding of isolated free fluid on abdominal CT alone is no longer an indication for laparotomy. Rodriguez *et al*. [1], reported that isolated free fluid was present in 463 (2.8%) of more than 16 000 blunt trauma patients studied; only 122 (27%) of these patients underwent therapeutic laparotomy.

Drasin *et al*. [ 2], conducted a study to determine the clinical significance of the isolated finding of free intraperitoneal fluid on MDCT in male patients who have suffered blunt trauma. The radiologists found free fluid in 48 patients (7.2%). Twenty-nine (4.3%) of these patients had one or more identifiable causes for the free fluid (liver injury, splenic injury, bowel injury, mesenteric hematoma, pancreatic fracture, extensive pelvic fractures or retroperitoneal injury without identifiable intraperitoneal injury, and isolated large-bowel injury). In the remaining 19 (2.8%), the intraperitoneal free fluid was an isolated finding with no identifiable injuries. Those 19 patients were discharged without any further abdominopelvic complaint or intervention.

On the significance of detecting intra-abdominal free fluid without solid organ injury on computed tomography in hemodynamically stable patients with blunt abdominal trauma, Mahmood *et al*. [3] conducted a retrospective analysis of 122 cases. They concluded that detecting intra-peritoneal fluid by computed tomogram scan is inaccurate for predicting bowel injury or the need for surgery. A small amount of isolated free fluid in the deep region of the pelvis in male with blunt trauma was identified in the MDCT scan. This was not considered as a sign of bowel and/or mesenteric injury according to Yu *et al.* [4] in their retrospective study of 1000 blunt abdominal trauma cases. To our knowledge, this problem was not previously addressed specifically in professional male football players.

## Conclusion

Isolated free intra-abdominal fluid on MDCT scan in previously healthy male patients with blunt trauma and no solid organ, bowel, and/or mesenteric injury may be of no clinical significance. However, further studies are needed to explain and support this finding.

## Author contributions

All authors have made a substantial contribution to the concept, design of the work; and interpretation of data, drafted the article and revised it critically for important intellectual content, and approved the version to be published.

## Ethical approval

The Medical Research Center of Hamad Medical Corporation has granted permission for this case report to be published on condition that no patient-identifiable data (including patient name and photograph) are included (MRC-04-23-432).

## Conflict of interest statement

None declared.

## Funding

None declared.

## Data availability

Data used to support the findings of this case are included within the article.
